# Hormonal and Molecular Regulation of Phallus Differentiation in a Marsupial Tammar Wallaby

**DOI:** 10.3390/genes11010106

**Published:** 2020-01-16

**Authors:** Yu Chen, Marilyn B. Renfree

**Affiliations:** 1 Department of Molecular Genetics and Microbiology, University of Florida, Gainesville, FL 32603, USA; 2 School of BioSciences, The University of Melbourne, Parkville, VIC 3010, Australia

**Keywords:** lncRNA, WGCNA, marsupial, androstanediol, RNAseq, *IGF1*, *SHH*, oestrogen, castration, phallus

## Abstract

Congenital anomalies in phalluses caused by endocrine disruptors have gained a great deal of attention due to its annual increasing rate in males. However, the endocrine-driven molecular regulatory mechanism of abnormal phallus development is complex and remains largely unknown. Here, we review the direct effect of androgen and oestrogen on molecular regulation in phalluses using the marsupial tammar wallaby, whose phallus differentiation occurs after birth. We summarize and discuss the molecular mechanisms underlying phallus differentiation mediated by sonic hedgehog (*SHH*) at day 50 pp and phallus elongation mediated by insulin-like growth factor 1 (*IGF1*) and insulin-like growth factor binding protein 3 (*IGFBP3*), as well as multiple phallus-regulating genes expressed after day 50 pp. We also identify hormone-responsive long non-coding RNAs (lncRNAs) that are co-expressed with their neighboring coding genes. We show that the activation of *SHH* and *IGF1*, mediated by balanced androgen receptor (AR) and estrogen receptor 1 (ESR1) signalling, initiates a complex regulatory network in males to constrain the timing of phallus differentiation and to activate the downstream genes that maintain urethral closure and phallus elongation at later stages.

## 1. Introduction

**Figure 1:**
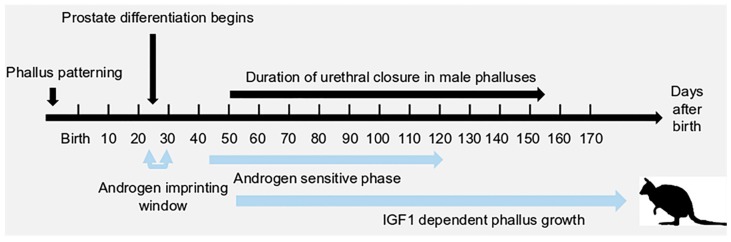
The timeline of prostate differentiation, phallus development, the androgen imprinting window, the androgen sensitive phase, and the insulin-like growth factor 1 (*IGF1*) dependent phase in the tammar wallaby.

The marsupial tammar wallaby has been used as a molecular research model to study sex determination and sexual differentiation for decades. It is a unique model to investigate sex-related molecular regulation due to its extended period of postnatal sexual differentiation. In the tammar, testicular differentiation occurs from two days after birth, while ovarian differentiation does not begin until day eight postpartum (pp) (reviewed in [1]). Although the genital tubercle is detected about two days before birth [2], sexually dimorphic phallus differentiation does not begin until day 50 pp [3] (Figure 1). After day 50 pp, the anogenital distance in males becomes longer than that in females [2,3,4,5]. The male phallus elongates faster and the urethra begins to fuse along the ventral midline, while the female urethra remains unfused [2,3,4,5]. By day 150 pp, the urethral meatus has reached the glan penis in males, whereas in females, the urethra remains open [3,5].

The development of male tammar phallus is androgen dependent [5,6], like that of eutherian mammals. In males, there is an increase of testicular testosterone from birth to day 40 pp [7]. However, testicular testosterone concentration falls sharply after day 40 pp, but is unmeasurable in ovaries, and plasma levels do not differ between the sexes up to day 50 [3,5,7,8]. There is a critical androgen imprinting window (window of androgen sensitivity or androgen programming window) between days 25 to 30 pp, first described in the tammar in 2004 and then identified in rats and humans [5,9,10,11,12]. Altering androgen concentrations by castration of male young or treatment of female young with the potent androgen androstanediol during this programming window contributes to abnormal phallus development, including hypospadias or phallus sex reversal [5,6]. Interestingly, although androgen controls both urethral closure and phallus elongation, the molecular regulation behind these two phases can differ. When males are castrated at day 25 pp, their phalluses are feminized and the urethra remains unfused [5]. When males are castrated at day 40 pp or at day 80 pp, their phalluses become shorter but the treatment has no effect on urethral closure [5]. These results indicate that urethral closure is regulated by the androgen priming, whereas phallus elongation requires constant or increasing levels of androgen. Although several phallus regulating genes in the tammar have been reported, there has been less attention on the signalling pathways during phallus development. 

In this paper, we review the molecular regulation of androgen priming on *sonic hedgehog* (*SHH*), *insulin-like growth factor 1* (*IGF1*) and long non-coding RNA (lncRNAs), during phallus differentiation in the tammar. First, we present the molecular mechanism that initiates the phallus differentiation at day 50 pp and, then, the regulatory mechanism of phallus elongation at day 90 pp. We also identify hormonal responsive lncRNAs during phallus development in the tammar and describe their relationship to their neighboring coding genes. 

## 2. A Unique Androgen-Sensitive Regulation Network of *Sonic Hedgehog (SHH)*

**Figure 2:**
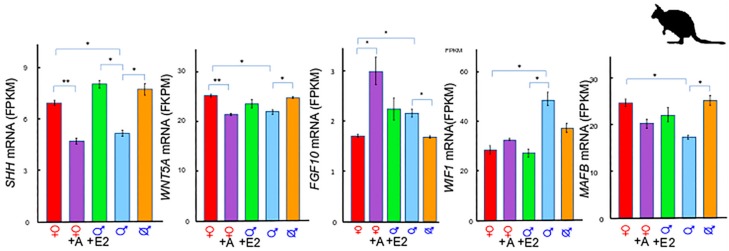
Gene expression of *SHH*, *WNT5A*, *MAFB*, *FGF10*, and *WIF1* in phalluses at day 50 pp. *SHH*, *WNT5A*, and *MAFB* expression is higher in female phalluses and increases in male phalluses after castration. *SHH* is upregulated by oestrogen treatment in males but downregulated in females after adiol treatment. *WNT5A* is downregulated in females after adiol treatment. Both *FGF10* and *WIF1* are higher in male phalluses at day 50 pp. *FGF10* is upregulated by adiol treatment and *WIF1* is downregulated by oestrogen treatment. A: adiol, E: oestrogen, *: *p*-value < 0.05, **: *p*-value < 0.005, SEM: Standard error of the mean (error bar), Red: control female phalluses, purple: adiol treated female phalluses, green: oestrogen treated male phalluses, blue: control male phalluses, and orange: male phalluses with testes removed (male symbol with a cross). Figure redrawn from [13,16].

In the tammar, *SHH* expression remains low in males when testicular testosterone is high, but increases after the content of testosterone (ng/mg protein) in the testes drops [7,9]. Similarly, in phallus transcriptome data (Figure 2), *SHH* expression increases after removing the testes, but decreases in female phalluses when given androgen [13]. The negative association between *SHH* expression and androgen is also seen in a lymph node carcinoma of the prostate (LNCaP) cell line [14]. Through steroid treatment and RNA-sequencing (RNA-seq) data analysis in the tammar, a number of genes are shown to have a similar expression pattern to that of *SHH*. *SHH*, *Wnt family member 5A* (*WNT5A*), and *MAF BZIP transcription factor B* (*MAFB*) are all downregulated in female phalluses by androgen treatment at day 50 pp, but are upregulated after castration in males [13,15], while *fibroblast growth factor 10* (*FGF10*) is upregulated by androgen treatment, but downregulated after castration in males [15]. 

### 2.1. SHH and WNTs

Like *SHH*, *WNT5A* is also downregulated by androgen treatment in females, but increases in males after castration in the tammar [13]. The interaction between androgen and activation of *SHH* and *WNT5A* can be critical to maintain masculinization of tammar phalluses, as seen in mice [17,18]. In *SHH* knockout mice, there is a decrease in proliferation and an increase in apoptosis [19], as well as decreases in *Wnt* gene expression and WNT/β-catenin signalling activity [18]. *WNT inhibitory factor 1* (*Wif1*) negatively regulates WNT/β-catenin signalling to balance cell apoptosis in mice [20,21,22]. In the tammar, WNT Inhibitory Factor 1(*WIF1*) is higher in male phalluses and is downregulated by oestrogen treatment, which is opposite to that of *SHH* [13]. This opposite expression pattern suggests that maintaining phallus development requires a balanced SHH signalling and WIF/WNT signalling in the tammar.

### 2.2. SHH and MAF BZIP Transcription Factor B (MAFB)

In the tammar, both *SHH* and *MAFB* are higher in normal female phalluses and are increased in phalluses after castration in males at day 50 pp [15]. This is in contrast to our expectation due to the predominant role of *Mafb* in male phalluses in mice [23,24,25]. It is likely that *MAFB* acts as a downstream target of SHH/WNT signalling in the tammar, as the gene is under the regulation of β-catenin, a transcription factor of the WNT pathway in mice [24]. More interestingly, unlike *SHH*, which is only transiently increased in male phalluses around day 50 pp [9], *MAFB* increases in normal male phalluses by day 90 pp [15]. This data suggests that *MAFB* could have a dual role at the early stage of phallus differentiation and at the later stage of phallus elongation.

### 2.3. SHH and Fibroblast Growth Factor 10 (FGF10)

In the tammar, *FGF10* expression is upregulated by androgen [15], unlike *SHH*, *WNT5A*, and *MAFB* that are downregulated [13,15]. In mice, high levels of *SHH* inhibits *FGF10* transcription in the endoderm during lung morphogenesis [26]. In the tammar, a transient high level of *SHH* in male phalluses at day 50 pp (mentioned above) may suppress *FGF10* expression. When *SHH* decreases after day 50 pp [9], *FGF10* increases [15]. Therefore, it is possible that SHH signalling suppresses *FGF10* expression at day 50 pp before phallus elongation. However, after day 50 pp, while *SHH* expression goes down, *FGF10* increases, presumably to maintain phallus elongation at later stages, as seen in mice [27,28,29].

### 2.4. The SHH Switch

*Sonic Hedgehog* is negatively regulated by androgen in the tammar, which is unusual as compared with eutherian mammals. *SHH* levels transiently increase when testicular testosterone drops at around day 40 pp [9]. After day 50 pp, there is no significant difference in plasma testosterone, plasma dihydrotestosterone, and adrenal testosterone between males and females up until day 150 pp [7,8]. However, there are increased levels of the potent androgen androstanediol [5,30] which appears to be critical to maintain phallus elongation and urethral closure after day 50 pp in the tammar. 

Sexually dimorphic structures differentiate post-natally in marsupials and over a long time period. Prostate differentiation in the tammar begins at day 25 pp in males [31], while the phallus does not become sexually dimorphic until day 50 to 60 pp. This is in marked contrast to humans, mice and rats in which phallus differentiation begins synchronously with prostatic, ductal, and testicular androgen production. During pregnancy in humans, the prostate and penis differentiate at about 10 weeks [32,33,34,35,36], at 16.5 to 17.5 days in mice [37,38,39,40,41], and at 17 to 19 days of gestation in rats [42,43]. The unique *SHH* increase might be a regulatory mechanism to constrain the onset of phallus dimorphism up to day 50 to 60 pp in the tammar and switch it on in the males at this time. 

SHH and IGF signalling have a synergistic relationship to induce proliferation in multiple tissues in mice [44,45,46]. In addition, *SHH*-induced proliferation is inhibited by the anti-IGFR1 blocking antibody, cixutumumab (IMC-A12) [44]. IGF2 binds to the IGFR1 [47], and since hepatic *IGF2* in the tammar is highest in males from day 50 to 70 pp [16], it may have a similar relationship with SHH signalling at days 50 to 60 pp in tammar phalluses to regulate *SHH*-induced proliferation. 

## 3. *Insulin-Like Growth Factor 1* (*IGF1*) in Phallus Growth and Urethral Closure

Laron syndrome (OMIM ID #262500), also known as growth hormone (GH) insensitivity syndrome, affects phallus growth and leads to micro-penis [48]. Without GH, IGF1 is not secreted at sufficiently high levels, so IGF1 treatment in human patients can reverse the micro-penis seen in Laron syndrome [49,50]. The lifespan of IGF1 and its pathway activity is affected by the insulin-like growth factor binding proteins (IGFBPs) [51,52,53]. However, the interplay between *IGF1* and *IGFBPs* in phallus development has not been thoroughly investigated in eutherian mammals. Here, we review the role of the IGF network by using RNA-seq analysis and co-expression analysis in phalluses with a tammar as a model. 

### 3.1. IGF1 and Insulin-Like Growth Factor Binding Protein 6 (IGFBP6)

**Figure 3:**
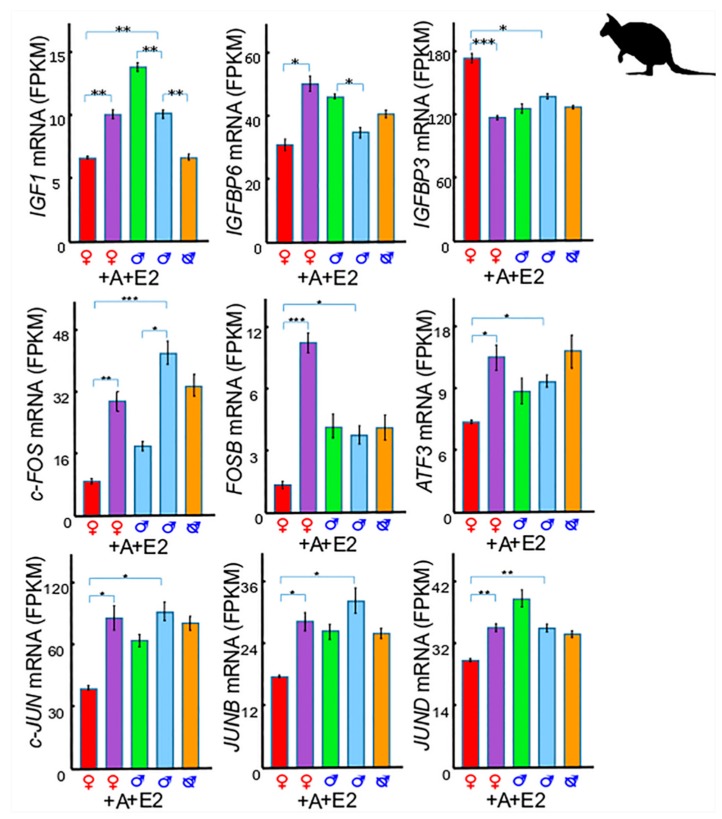
Gene expression of *IGF1*, *IGFBP3*, *IGFBP6*, and Activator Protein 1 (AP-1) genes in phalluses at day 50 pp. Both *IGF1* and *IGFBP6* are upregulated by adiol and oestrogen treatment. *IGF1* is higher in normal male phalluses at day 50 pp and is downregulated in males after castration. *IGFBP3* is higher in female phalluses at day 50 pp and is downregulated in females after adiol treatment. All six AP-1 genes (*C-FOS*, *FOSB*, *ATF3*, *c-JUN*, *JUNB*, and *JUND*) are higher in male phalluses and upregulated by adiol treatment. *C-FOS* is downregulated by oestrogen treatment. A: adiol, E: oestrogen, FPKM: Fragments per kilobase million; *: *p*-value < 0.05, **: *p*-value < 0.005, ***: *p*-value < 0.001. Figure redrawn from [15].

Both *IGF1* and *IGFBP6* are upregulated by androgen and oestrogen treatment in tammar phalluses [15] (Figure 3). Such androgenic and oestrogenic dependency of *IGF1* is also seen in eutherian mammals. For instance, testosterone increases *IGF1* in bovine muscle satellite cells [54], rat uterine tissue [55], and human prostate cancer cell lines [56,57]. Oestrogen also increases *IGF1* expression in the primate cerebral cortex [58] and in the mouse uterus [59]. Similarly, *IGFBP6* decreases in rat epididymides after blocking dihydrotestosterone (DHT) synthesis [60] and is upregulated in prostate cancer cells after treatment with diethylstilbestrol (DES), a synthetic oestrogen [61]. However, the detailed mechanisms remain unknown.

### 3.2. IGF1 and Insulin-Like Growth Factor Binding Protein 3 (IGFBP3)

In contrast to *IGF1*, *IGFBP3* expression is higher in female phalluses than male phalluses at day 50 pp, day 90 pp, and at day 150 pp, and is downregulated in female phalluses after androgen treatment [15] (Figure 3). A similar response is also found in eutherian mammals in which *IGFBP3* is downregulated in prostate cancer cells after treatment of androgen [57,62,63] or synthetic androgen [63]. The opposing expression of *IGF1* and *IGFBP3* suggests that *IGFBP3* may be the agent that inhibits female phallus development by negative regulation of cell proliferation, as seen in many other studies [57,64,65,66,67,68]. Thus, *IGF1* may be responsible for maintaining normal male phallus growth at later stages.

### 3.3. IGF1 and Activator Protein 1 (AP-1)

The transcription of *IGF1* is regulated by the DNA binding of Activator Protein 1 (AP-1) complex [69]. Interestingly, both *IGF1* and AP-1 genes are higher in males and increase in female phalluses after androgen treatment in the tammar [15] (Figure 3). Similar androgen sensitivity is also seen in other studies. For example, *Fos proto-oncogene*, AP-1 transcription factor subunit (c-Fos), and activating transcription factor 3 (*ATF3*) are induced by androgen in the rat hippocampus [70] and in human prostate cancer cells [71], respectively. Since androgen treatment induces phallus elongation [5] and urethral closure [13] in the tammar, it is likely that the AP-1 genes, under the regulation of androgen control cell proliferation in phalluses, as it does in other cells (reviewed in [72]). 

### 3.4. IGF1 and Urethral Closure

**Figure 4:**
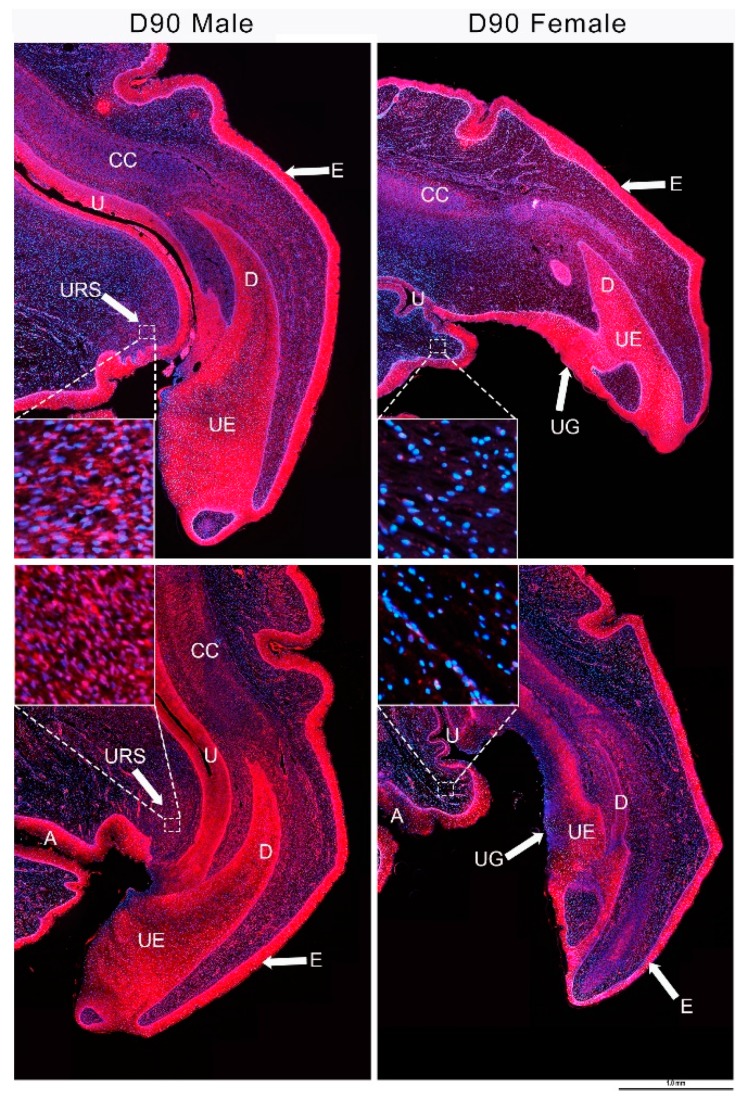
IGF1 and proliferating cell nuclear antigen (PCNA) distribution in phalluses at day 90 pp. In both male and female phalluses, IGF1 (**top**) and PCNA (**bottom**) are expressed in epithelial cells and in the corpora cavernosa. However, IGF1 and nuclear PCNA are found only in the URS of male phalluses (see insets). CC: corpus cavernosum, D: diverticulum, E: epithelium, U: urethra, UE: urethral epithelium, UG: urethra groove, URS: urorectal septum, red staining: IGF1 (**top**) and PCNA (**bottom**), and blue staining: DAPI (4′,6-diamidino-2-fenilindol). Scale bar, 1.0 mm. Figures from [15].

Several hypotheses are proposed to explain the mechanism of urethral closure. One of the hypotheses is that the proliferation of cells in urorectal septum (URS) contributes to the urethral closure [73,74,75]. Interestingly, IGF1 is localized in the mesenchyme of the URS only in male phalluses at day 90 pp, but is absent in that of female phalluses [15] (Figure 4). Proliferating cell nuclear antigen (PCNA), a marker for cell proliferation, shows similar localization [15] (Figure 4). The importance of *IGF1* at this time is further supported by an earlier study in the tammar that shows that hepatic expression of *IGF2* is significantly higher in males than females at day 70 pp, about the time that male and female phalluses become sexually dimorphic [16]. While *IGF2* decreases from day 70 and is no longer sexually dimorphic by day 100, hepatic and plasma levels of IGF1 significantly increase in both sexes from day 90 pp to day 250 of pouch life [16]. These data suggest for the first time that urethral closure may involve IGF1-mediated cell proliferation specifically in male URS. 

### 3.5. IGF1 Dependent Phallus Growth

A study conducted by Leihy et al., 2004 demonstrated for the first time an androgen sensitive phase during phallus elongation between days 20 and 40 pp in the tammar [5]. Removing testes in males before day 120 pp reduced phallus length while applying androgen treatment in females before day 120 pp enhances phallus elongation, but has no effect on urethral closure [5]. However, as mentioned before, there is no significant difference in plasma testosterone between male and female at least up to day 50 pp [7,8]. Thus, there must be another regulatory network that is activated by the earlier androgen window of sensitivity to maintain the phallus elongation after day 50 pp. SHH appears to be the key switch that initiates the expression of potential regulatory genes. These may include *IGF1*, *IGFBP3*, *FGF10*, *fibroblast growth factor receptor 2* (*FGFR2IIIb*), *Eph-related receptor tyrosine kinase ligand 5* (*EFNB2*)*, MAFB,* and *distal-less homeobox 5* (*DLX5*). The balance between *IGF1* and *IGFBP3* could be important in regulating phallus elongation and urethral closure. *FGF10*, *FGFR2IIIb*, *EFNB2*, *MAFB*, and *DLX5* may also involve phallus elongation, since they are significantly higher in male phalluses at day 90 pp [15] and have a conserved localization in urethral epithelium, as seen in mice [28,37,38,76,77,78]. In addition, these genes appear to be important to maintain cell proliferation and survival [79,80,81,82,83,84,85] during male phallus development in mice [23,29,86,87]. 

## 4. Co-Expression Network and Hormonally Responsive Long Non-Coding RNAs

Our RNA-seq dataset consists of five different treatment groups with 5 replicates for each group, which makes it hard to interpret with differential expression (DE) analysis. We used weighted genome co-expression network analysis (WGCNA) to find co-expressed genes. It is also a good way to identify lncRNAs as most of them have extremely low sequence conservation, making them difficult to identify cross species with alignment. In our previous paper, we set up a pipeline by combining WGCNA, DE analysis, and the location of lncRNAs and identified the following three coding gene-neighboring lncRNAs: *lnc-RSPO4*, *lnc-BMP5*, and *lnc-ZBTB16* [88].

### 4.1. IGF1, Androgen Receptor (AR), and ESR1 Co-Expression Network

**Figure 5:**
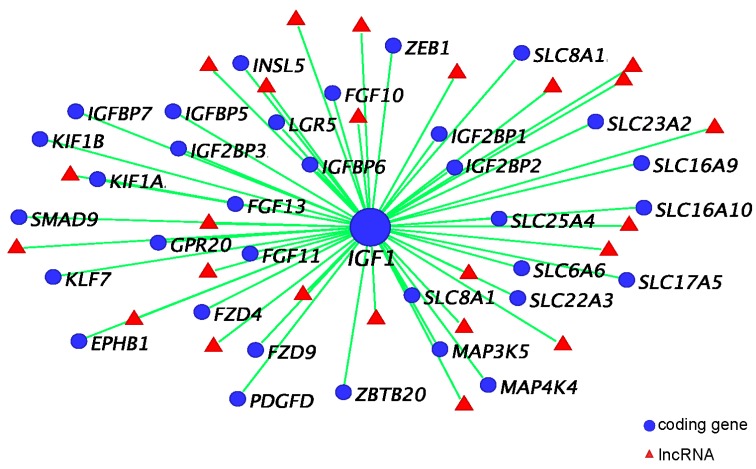
*IGF1*, androgen receptor (*AR*), and *ESR1* co-expression network. *IGF1* co-expressed coding genes (selected based on correlation and reference review) and predicted co-regulatory long non-coding RNAs (R > 0.9). Figure redrawn from [88].

*IGF1* is considered as a hub gene in its co-expression network due to its high correlation with a large number of protein-coding genes and lncRNAs. Within the *IGF1* co-expression network, both *IGFBP5*, an IGF signalling regulator (reviewed in [89]) that inhibits SHH-induced proliferation in cerebellar granule cells in mice [44], and *FGF10*, a phallus regulating gene in mice and the tammar [15,28,29,77,78], have a high correlation (R ≥ 0.8) with *IGF1* [88] (Figure 5). *IGF1* is also co-expressed with multiple genes that may have a role in regulating reproductive development (Figure 5). For instance, it is co-expressed with other IGF family members, including *insulin like growth factor 2 binding protein* (*IGF2BP*) 1–3, *insulin like 5* (*INSL5*), and *IGFBP7*. Apart from *FGF10*, *IGF1* is also associated with *FGF11*, *FGF13*, and *tyrosine-protein kinase receptor EPH-2* (*EPHB1*). Two receptors, *frizzled class receptor 4* (*FZD4*) and *FZD9*, in the WNT signalling pathway show high association with *IGF1*. Interestingly, *IGF1* is co-expressed with *zinc finger* and *BTB domain containing 20* (ZBTB20), whose mutation causes micro-penis [90]. We also find mutations of several *kinesin family members* (KIF), such as *KIF1A*, *KIF1B* and *KIF7*, that are associated with *IGF1* and can also induce an abnormal phallus phenotype in human [91,92]. These data further confirm the importance of *IGF1* in regulating phallus development in the tammar.

In mice, oestrogen signalling clearly has a regulatory role in phallus development [93,94], as we have found in the tammar [13,15,88]. In the tammar co-expression network, about 50% of estrogen receptor 1 (*ESR1*) co-expressed coding genes and lncRNAs are also associated with *AR* [88], suggesting an interaction between androgen receptor (AR) signalling and ESR1 signalling during tammar phallus development [88]. However, those lncRNAs could have other genetic targets because none of them were located within 100 kb upstream or downstream of *IGF1*, *AR*, and *ESR1*.

### 4.2. lnc-RSPO4, lnc-BMP5, and lnc-ZBTB16

We identified three novel lncRNAs using our pipeline. *Lnc-RSPO4* is co-expressed with roof plate-specific spondin-4 (*RSPO4*) coding gene. Both *RSPO4* and lnc-*RSPO4* are downregulated in tammar female phalluses after androgen treatment [88], in a similar expression pattern to that of *SHH* and *WNT5A* [13]. Interestingly, RSPO4 is a ligand of leucine-rich repeat containing G protein-coupled receptor (LGR) 4–6 receptors that potentiate WNT signalling [95,96,97,98]. Thus, *RSPO4* and *lnc-RSPO4* could also be involved in the molecular regulation mediated by *SHH* and *WNT5A* signalling during tammar phallus development.

Both *lnc-BMP5* and *lnc-ZBTB16* are downregulated in tammar male phalluses after oestrogen treatment [88]. They are also co-expressed with *bone morphogenetic protein* (*BMP5*) and *zinc finger and BTB domain containing 16* (*ZBTB16*), respectively, in our co-expression network [88]. Interestingly, *Bmp5* is downregulated by flutamide, an androgen signalling inhibitor during phallus development in mice [93]. Mutation of *ZBTB16* induces micro-penis [99,100], which is similar to the phenotype observed after oestrogen treatment in the tammar [13]. These data show that there is a complex regulatory system of lncRNAs during phallus development mediated by hormonal signalling. 

## 5. Conclusions and Future Directions

**Figure 6:**
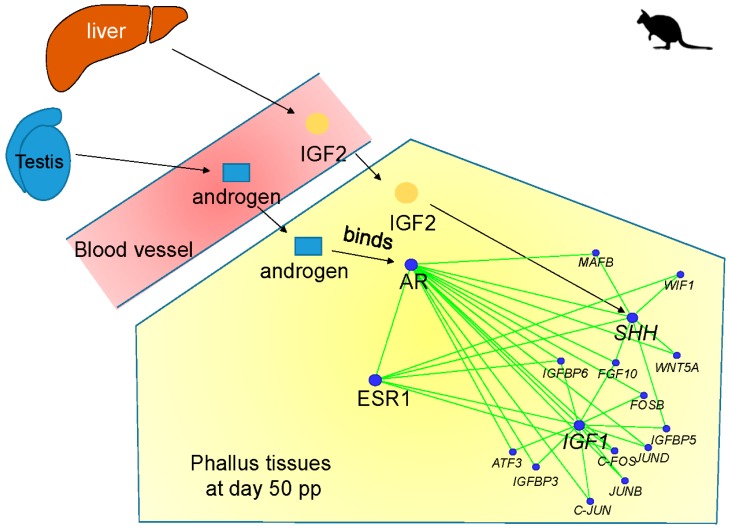
Summary of IGF2-SHH and androgen-IGF1 signalling networks. The activation of IGF2-SHH and androgen-IGF1 signalling networks initiate urethral closure in males, whereas in females, non-activation of those two signalling networks results in an unfused urethra.

Tammar phallus development is under the regulation of a complex molecular network mediated by endocrine hormones. This review describes two endocrine-mediated networks, the *SHH* network and the *IGF1* network, which may act as molecular switches to constrain and decide male phallus development (Figure 6). The RNA-seq analysis identifies two sets of genes, including *WNT5A*, *MAFB*, *RSPO4*, *lnc-RSPO4*, *FGF10*, *WIF1* and *AP-1*, *FGF10*, *IGFBP3*, *IGFBP6*, *IGFBP5*, *EFNB2*, that interact with *SHH* and *IGF1*, respectively, in the tammar phalluses at day 50 pp. Interestingly, due to the negative association between androgen and *SHH* transcription, an *SHH* switch could be a unique regulatory mechanism in the tammar to constrain the timing of phallus differentiation. 

The molecular regulatory network that maintains phallus growth after day 50 pp consists of another set of genes, including *IGF1*, *IGFBP3*, *FGF10*, *FGFR2IIIb*, *EFNB2*, *MAFB*, and *DLX5*. The increased level of those genes may be initiated and enhanced by activation of two endocrine-mediated SHH and IGF1 switches in males, resulting in a phallus with complete urethral closure and elongated shaft. In addition, it is likely that urethral closure is mediated by the activation of IGF1 signalling in the male urorectal septum.

Co-expression analysis to identify novel hormone-responsive lncRNAs, such as *lnc-BMP5*, *lnc-RSPO4*, and *lnc-ZBTB16*, in the tammar phalluses reveals complex regulatory networks of *IGF1*, *AR,* and *ESR1* that associate with multiple hormone-responsive coding genes and lncRNAs during tammar phallus development. The data also indicate a potential interplay between AR and ESR1 signalling.

Taken together, the activation of the SHH switch and IGF1 switch, mediated by the balance between AR and ESR1 signalling, initiate a complex regulatory network in males to constrain the timing of phallus differentiation and to activate the downstream genes that maintain urethral closure and phallus elongation at later stage.
